# Impacts of biodiversity and biodiversity loss on zoonotic diseases

**DOI:** 10.1073/pnas.2023540118

**Published:** 2021-04-05

**Authors:** Felicia Keesing, Richard S. Ostfeld

**Affiliations:** ^a^Program in Biology, Bard College, Annandale, NY 12504;; ^b^Cary Institute of Ecosystem Studies, Millbrook, NY 12545

**Keywords:** biodiversity, disease, disease ecology, zoonotic disease, zoonoses

## Abstract

Zoonotic diseases are infectious diseases of humans caused by pathogens that are shared between humans and other vertebrate animals. Previously, pristine natural areas with high biodiversity were seen as likely sources of new zoonotic pathogens, suggesting that biodiversity could have negative impacts on human health. At the same time, biodiversity has been recognized as potentially benefiting human health by reducing the transmission of some pathogens that have already established themselves in human populations. These apparently opposing effects of biodiversity in human health may now be reconcilable. Recent research demonstrates that some taxa are much more likely to be zoonotic hosts than others are, and that these animals often proliferate in human-dominated landscapes, increasing the likelihood of spillover. In less-disturbed areas, however, these zoonotic reservoir hosts are less abundant and nonreservoirs predominate. Thus, biodiversity loss appears to increase the risk of human exposure to both new and established zoonotic pathogens. This new synthesis of the effects of biodiversity on zoonotic diseases presents an opportunity to articulate the next generation of research questions that can inform management and policy. Future studies should focus on collecting and analyzing data on the diversity, abundance, and capacity to transmit of the taxa that actually share zoonotic pathogens with us. To predict and prevent future epidemics, researchers should also focus on how these metrics change in response to human impacts on the environment, and how human behaviors can mitigate these effects. Restoration of biodiversity is an important frontier in the management of zoonotic disease risk.

## A Confusing Role for Biodiversity in Pathogen Transmission?

Thousands of pathogens circulate in the human population; hundreds of these are bacteria (1), hundreds more are viruses (2); a smaller but still sizeable number are fungi (3). Many of these infectious agents circulated first in other vertebrate animals, such as mammals and birds. In their original host species, the microbes might have lived without harming their hosts, or they might have caused disease. Regardless, at some point they spilled over into humans and began causing illness.

The transfer of microbes from animals to humans has occurred across millennia. Some of these microbes caused the scourges of our ancestors, from plague to smallpox to tuberculosis (4). More recently, humans have confronted AIDS, Ebola, severe acute respiratory syndrome (SARS), and Middle East respiratory syndrome (MERS). These so-called zoonotic diseases, which result from cross-species transmission of pathogens between humans and other vertebrate animals, appear to be emerging more frequently (5). Certainly, the COVID-19 pandemic has made the risks of zoonotic diseases a vivid and harrowing reality for every person on Earth.

Until recently, habitats with naturally high levels of biodiversity were thought to serve as hotspots for the emergence of new zoonotic pathogens, presenting a hazard to humans (5, 6). This expectation was based on the assumptions that a diversity of free-living organisms leads to a diversity of pathogens, and that pathogen diversity per se is a risk factor for zoonotic emergence (7). But for decades, we have also known that under some conditions, high biological diversity can decrease the transmission of zoonotic diseases that have already become established (8, 9). Taken together, these conflicting findings appeared to mean that the loss of natural biodiversity could simultaneously increase human exposure to existing pathogens, and decrease the probability of the emergence of new ones. Such a potential contradiction has complicated the ability of scientists to provide useful information about diversity–disease relationships for public policy and management.

Here, we evaluate recent evidence indicating how biodiversity affects both the emergence of new zoonotic diseases and the transmission of established ones. We first explore the effects of overall biodiversity, versus the biodiversity of particular taxa, on the emergence of zoonotic pathogens. We then review recent studies addressing whether some taxa are more likely to serve as sources of zoonotic pathogens. We consider how changes in biodiversity, especially changes arising from anthropogenic impacts, affect community composition relevant to disease dynamics. Finally, we evaluate whether recent evidence allows the effects of biodiversity, particularly its loss, on pathogen emergence to be reconciled with their effects on subsequent transmission.

## Biodiversity as a Source of Zoonotic Pathogens

Animals share their pathogens with us the same ways that humans share their pathogens with each other. A pathogen might travel from one host to another in droplets or aerosols from coughs or sneezes; through blood, urine, saliva, or other bodily fluids; through fecal material; or by being transferred during the bite of a vector like a fly, mosquito, or tick. In some cases, the pathogen might linger on a surface or in the environment so that a human might encounter the pathogen without close proximity to the animal that was its source. The pathogen might not be able to infect the human it contacts. Even if it can, the person’s immune system might stop the pathogen before it causes harm. But in some cases, the pathogen is able to infect the new human host, and that person might in turn transmit the pathogen to others.

What factors determine whether a pathogen will spill over from an animal into a human host and become established? Cross-species transmission results from a complex interplay between the characteristics of the pathogen (2, 10–12): the original host’s infection, behavior, and ecology; how the pathogen is shed into and survives in the environment; how humans are exposed to the pathogen; and how susceptible those humans are to infection (4, 12–16).

**Fig. 1.**
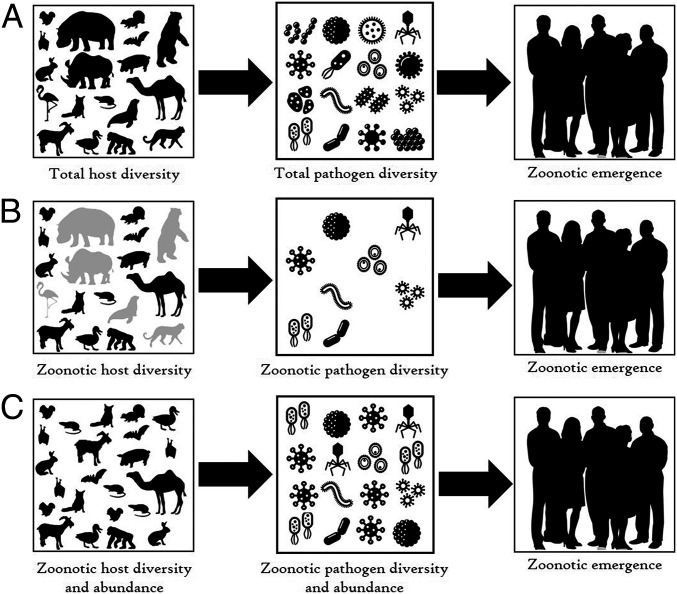
Alternative conceptual models linking host biodiversity to zoonotic emergence in humans. (*A*) Total host diversity: In this model, the overall diversity of hosts leads to a pool of pathogens, any one of which could jump to humans. Research assuming this model typically involves comparisons of large geographic areas with innate variation in biodiversity (e.g., along latitudinal gradients or between countries). (*B*) Zoonotic host diversity: In this model, some species are more likely to host zoonotic pathogens, and it is the diversity of these zoonotic hosts that is most important in determining the risk of zoonotic emergence. Research using the zoonotic host diversity model typically focuses on the distribution or characteristics of a particular taxon (e.g., bats or primates). (*C*) Zoonotic host diversity and abundance: In this model, the diversity and the abundance of zoonotic hosts determine the risk of zoonotic emergence. Research using this model typically focuses on the effects of changes in natural biodiversity (e.g., through human impacts, on zoonotic pathogens). Modified from an illustration in Ostfeld and Keesing (7).

Natural biodiversity, and its loss, can affect this pathway at multiple points, potentially affecting the probability that a new pathogen will become established in humans. Most importantly, diverse communities of host species can serve as sources for new pathogens, and it is this role for biodiversity that has received the most attention in research on disease emergence. In the most common conceptual model linking biodiversity to disease emergence, biodiversity is made up of species that host a diversity of pathogens (*SI Appendix*), any one of which could have the characteristics enabling it to jump successfully into humans (Fig. 1*A*) (7). Implicit in this model focusing on total host diversity is the assumption that all taxa are equally likely to be sources of zoonotic pathogens. Alternatively, certain groups—such as bats, rodents, or livestock—might be significantly more likely to serve as sources of zoonotic pathogens. In this “zoonotic host diversity” model, the diversity of these hosts, but not total host biodiversity, would be most important in determining the probability of zoonotic emergence (Fig. 1*B*).

Researchers explicitly or implictly applying the “total host diversity” model (Fig. 1*A*) tend to conduct broad geographic comparisons across regions that differ in their innate levels of biodiversity. For example, in a seminal study, Jones et al. (5) identified zoonotic diseases that had emerged between 1940 and 2005, and mapped the most likely locations of their underlying emergence. After attempting to correct for potential spatial variation in reporting bias, Jones et al. compared a suite of variables to see which best predicted the locations of global zoonotic hotspots. Although zoonotic diseases arising from wildlife were only ∼1% more likely to emerge where the diversity of wild mammals was high, Jones et al. (5) concluded that “wildlife host species richness is a significant predictor for the emergence of zoonotic EIDs [emerging infectious diseases] with a wildlife origin, with no role for human population growth, latitude or rainfall.” Of note was their observation that high human population density increased the likelihood of the emergence of a zoonotic disease from wildlife by 75 to 90%, an effect almost two orders-of-magnitude greater than the effect of mammalian diversity. Allen et al. (17) expanded this analysis, incorporating more explanatory variables and new methods for estimating reporting bias. After correcting for reporting bias, they found that mammal species richness had only the fourth strongest influence on the distribution of emerging infectious diseases, after the presence of evergreen broadleaf trees first, human population density second, and climate third.

A study by Pedersen and Davies (18) exemplifies research underlain by the “zoonotic host diversity” model (Fig. 1*B*), in which some taxa are expected to more frequently be sources of zoonotic pathogens. Pedersen and Davies focused on primates. They divided the process of spillover into a new host species into three steps—opportunity, transmission, and establishment—each of which has specific drivers. Their first step, the opportunity for transmission, is underlain by the biogeography of host and pathogen. Their analysis rested on the assumption that step 3—establishment—is critical, and that it is strongly affected by ecological and evolutionary barriers between the current host species and a new host species. For this reason, they assumed that host species that are more closely related to humans will be the most likely sources for pathogens that can become zoonotic. Thus, Pedersen and Davies focused on primates, categorizing the risk of zoonotic spillover based on phylogenetic relatedness and geographic co-occurrence of primates worldwide. They found a hotspot for probable zoonotic spillover in central and western Africa, for example, because there is broad geographic overlap between humans and primate species to which humans are particularly closely related. Identifying the geographic locations or characteristics of taxa most responsible for zoonotic pathogens has been a focus of many recent studies (e.g., refs. 10, 11, and 19–23).

In the “zoonotic host diversity and abundance” model, both the diversity and the abundance of the animals most likely to act as hosts for zoonotic pathogens are critical (Fig. 1*C*). Thus, both the “zoonotic host diversity” and “zoonotic host diversity and abundance” models (Fig. 1 *B* and *C*) rely on weighting the importance of particular components of biodiversity by their zoonotic potential. To address whether this additional information is essential, we next review evidence addressing whether some taxa are more likely than others to serve as sources of zoonotic pathogens.

## Are Some Taxa More Likely to Transmit Zoonotic Pathogens?

The identity of the taxa most likely to serve as sources of zoonotic pathogens has been a major area of research. Using a database of ∼800 zoonotic pathogens, for example, Woolhouse and Gowtage-Sequeria (24) identified “ungulates” (a paraphyletic grouping that includes hooved mammals from two mammalian Orders) and Carnivores as the sources of the greatest numbers of zoonotic pathogens, with bats hosting the fewest. At about the same time, Dobson (25) and Calisher et al. (26) highlighted the importance of bats, a taxon that has been the focus of many subsequent studies (11, 27). In recent analyses, rodents have also emerged as the most likely source (21, 23) or one of the most likely (11, 27).

**Fig. 2.**
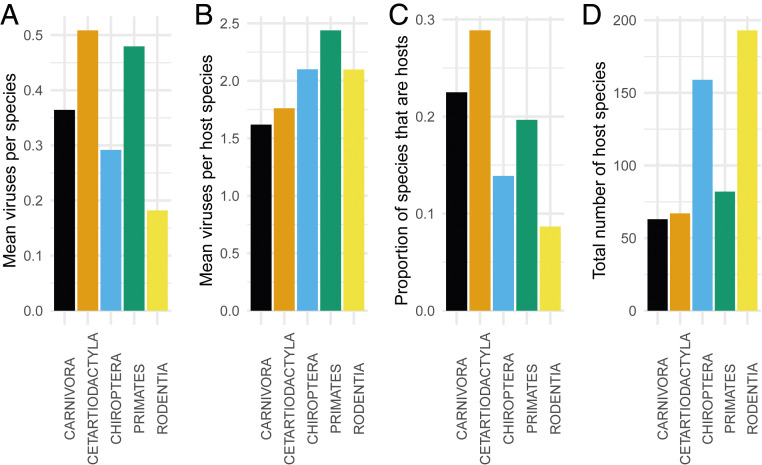
Relative importance of five major mammalian Orders as hosts of zoonotic viruses based on different metrics. (*A*) Mean number of viruses per host for all species in the Order. (*B*) Mean number of viruses per host for species that host at least one virus. (*C*) Proportion of all species that host at least one virus. (*D*) Total number of species in the Order that host at least one virus. The variety of metrics used in different studies is a source of confusion in competing claims about taxonomic importance. Plotted from data made available in the supplemental material from Johnson et al. (21); see caveats about these and similar data in *SI Appendix*.

Why does it matter whether we can identify certain taxa as more likely zoonotic sources than others? Such knowledge might narrow research focus from studies of total biodiversity to more relevant studies on specific taxa, thereby allowing targeted surveillance of particular high-risk groups or locations. For example, Han et al. (22) identified traits associated with rodents that are zoonotic hosts compared to rodents that are not, leading to predictions about particular rodent species that might harbor undetected zoonotic pathogens. Such knowledge might also provide important insights about policy or management (Fig. 2).

Recent work on animal sources of zoonotic pathogens has focused on viruses because these have been identified as the pathogens most likely to cause emerging zoonotic diseases (5). Johnson et al. (21) compiled a database of 142 zoonotic viruses and determined that the Order Rodentia is the source for two-thirds of the viruses originating from mammals, more than any other Order. From this analysis, bats (Order Chiroptera) host the second greatest number of viruses, with Carnivora (e.g., dogs and cats), Cetartiodactyla (mostly hooved mammals like sheep, cows, and deer), and Primates having comparatively high numbers of viruses relative to their diversity (but see Fig. 2). Mollentze and Streicker (28) compiled a larger database of viruses that are both zoonotic and nonzoonotic, and that infect both mammals and birds. They concluded that mammalian and avian Orders have the number of zoonotic viruses that would be expected based on each group’s share of diversity, and that no special characteristics of a group (e.g., immunological traits) need to be invoked to explain a group’s zoonotic contributions. Like Johnson et al. (21), Mollentze and Streicker (28) identify rodents as the group hosting the greatest number of zoonotic viruses.

An important theme about zoonotic hosts has been the role of domesticated species. For example, domesticated species have been proposed to be optimal “bridge hosts” (in the sense of refs. 29 and 30) for zoonotic pathogens, meaning that they can acquire pathogens from wild hosts that they then transmit to humans through proximity, density, and contact frequency. Including variables to attempt to account for reporting bias, Johnson et al. (21) found that domesticated species from across mammalian Orders, especially Cetartiodactyla and Carnivora, hosted on average 100 times as many viruses per species as their wild counterparts did. Wells et al. (31) used a more expansive definition of domesticated animals that included common commensal rodent species, such as house mice (*Mus musculus*) and rats (*Rattus norvegicus*, *Rattus rattus*). They included both viruses that are known to be zoonotic (*n* = 138 viruses) and those that are not (*n* = 1,647). Based on patterns of shared viruses, domesticated species had significantly higher centrality—an index of the degree to which that species is connected to other host species—than wild species did. Wardeh et al. (19) found that domestication status was a strong predictor of whether a species shares pathogens with humans. Johnson et al. (32) came to a different conclusion about the role of domesticated species, concluding that wild species were significantly more likely to have been the source of spillover events. Rodents, for example, were determined to be the source for 58% of the 95 zoonotic viruses in their analysis.

Although different research groups draw different conclusions regarding which vertebrate taxa are more likely to transmit pathogens to humans, the evidence for unequal impacts among the taxonomic groups is strong. Five Orders of mammals (Primates, Cetartiodactyla, Carnivora, Rodentia, and Chiroptera) are the most common sources. This evidence strongly reduces the appropriateness of the “total host diversity” model and increases that of the two models that focus on zoonotic host diversity (Fig. 1).

Johnson et al. (21) and Wells et al. (31) conducted their analyses with data that included hosts known to have been infected with a particular virus, and for which there was some evidence that they could share the pathogen with humans (*SI Appendix*). However, they did not attempt to identify the species that served as the original transmitter of the pathogen to humans: that is, the source of the primary spillover event that first resulted in a human infection. Instead, they focused on secondary spillover to humans, which can occur when the original host of the pathogen transmits to another host, which then transmits infection to humans, or when there is reciprocal transmission between humans and other animals. Making a distinction between primary and secondary spillover is difficult. Most pathogens that spill over to humans have broad host ranges (24, 30, 32, 33), so identifying a single species or taxon as the primary source is problematic. In practice, the primary source of a zoonotic pathogen is rarely identified definitively. For example, the primary source of SARS-CoV-2, which causes COVID-19, has not been identified. Relatives of the virus, with genetic similarities to SARS-CoV-2 in the high 90% range, have been found in horseshoe bats and pangolins (34, 35), but the only nonhuman animals currently known to host SARS-CoV-2 are those to which humans have transmitted it, either intentionally or unintentionally. These species include tigers (*Panthera tigris*), lions (*Panthera leo*), minks (*Neovison vison*), rhesus macaques (*Macaca mulatta*), and Siberian hamsters (*Phodopus sungorus*) (34, 36). Of these, at least minks appear to be able to transmit SARS-CoV-2 back to humans (37), so they could be considered a secondary spillover host, but they were not the primary spillover host. Most analyses of spillover focus on secondary spillover hosts like minks rather than primary spillover hosts, though this distinction is rarely explicit.

## How Human Impacts Influence Zoonotic Hosts

Human impacts like land-use change have been linked to emerging infectious diseases of humans in many studies (e.g., refs. 5, 8, 29, 38, and 39). Murray and Daszak (38), for example, explored how land-use changes, like deforestation and agricultural conversion, could affect the emergence of zoonotic viruses and presented two hypotheses. In one, land-use change increases contact between humans and a pool of diverse pathogens, without directly affecting the pool of pathogens. In the other, land-use change perturbs ecological communities, affecting zoonotic host species, such as rodents or bats, resulting in changes to cross-species transmission rates. These hypotheses are not mutually exclusive. Species that thrive in human-impacted habitats could provide opportunities for spillover based on both the diversity of their potential pathogens and their abundance, which might result in greater contact with humans (Fig. 1*C*). Simultaneously, human activity in these altered habitats could affect contact rates.

**Fig. 3.**
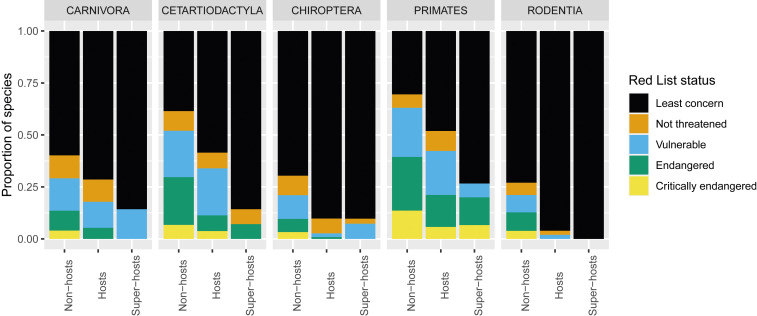
Proportion of species in each conservation category for nonhosts, hosts, and superhosts in the five Orders of mammals that host the majority of zoonotic viruses. “Non-hosts” harbor no known zoonotic viruses, “Hosts” harbor one to two, and “Super-hosts” harbor three or more. For all five Orders, hosts and superhosts are more likely to be in the conservation category of least concern. Plotted from data made available in supplementary materials from Johnson et al. (21); see caveats about these and similar data in *SI Appendix*. Species for which data needed to assign a conservation status were unavailable have been excluded.

Johnson et al. (21) found that 11% of 5,335 wild terrestrial mammal species were hosts of zoonotic viruses and most of these hosted only one such virus. Species that host zoonotic pathogens were more likely to be of lower conservation concern (e.g., they were more abundant) than species that do not (Fig. 3). Their results suggest that zoonotic host status in mammals may be positively correlated with resilience to human impacts, such as land conversion, direct exploitation (e.g., hunting, trade), pollution, and the spread of invasive species.

Gibb et al. (40) directly analyzed the effect of human impacts on host diversity and abundance. By combining multiple databases, they compiled a catalog of 6,801 ecological assemblages and 376 host species to ask whether zoonotic host species were more diverse or abundant, or both, in habitats intensively used or managed by humans. After controlling for research effort, they found that wild species known to be zoonotic hosts were more abundant and more diverse (as measured by species richness) in human-impacted habitats compared to less disturbed habitats. In contrast, wild species not known to be zoonotic hosts declined in diversity and abundance in human-impacted habitats. Mendoza et al. (41) came to a similar conclusion using a smaller dataset of ecological communities and zoonotic hosts.

Because the evidence linking hosts and pathogens in Gibb et al. (40) varied in quality, they reran their analyses on mammals using only host–pathogen associations for which they had a more rigorous metric, such as PCR detection of the pathogen or known reservoir status. Their conclusions remained unchanged.

Gibb et al. (40) provide evidence that the diversity and abundance of animals in human-impacted habitats shifts toward species that are more likely to be competent zoonotic hosts (*SI Appendix*). There is less evidence evaluating the effect of host abundance on emergence, but some studies suggest abundance is a key factor (e.g., ref. 42). The “zoonotic host diversity and abundance” model thus appears to be more realistic than the model that considers only “zoonotic host diversity”, and it is far more appropriate than the “total host diversity” model (Fig. 1).

## Reconciling the Role of Biodiversity in Emergence and Transmission

The analyses by Gibb et al. (40) and Johnson et al. (21) set the stage for a new understanding of the role of biodiversity, and changes to biodiversity, in the emergence and transmission of zoonotic diseases. Two decades ago, we proposed that innate biodiversity can reduce the risk of infectious diseases through a dilution effect, in which species in diverse communities dilute the impact of host species that thrive when diversity declines (43). In the years since, this phenomenon has been explored, debated, and reviewed (8, 9, 44–47), its mechanisms delineated (48) and explored (49–52), and its most basic principles regularly reexamined (53).

The dilution effect occurs when the transmission of a pathogen (*SI Appendix*) increases as diversity declines, as has been demonstrated for a number of disease systems. For example, in a series of comparative and experimental studies, Pieter Johnson and his colleagues (54, 55) have shown that the most competent reservoir species for a trematode parasite of amphibians, *Ribeiroia ondatrae*, is the Pacific tree frog, *Pseudacris regilla*. The frogs are also the species most likely to thrive as diversity declines in the ponds in which they live, which results in increased transmission of the parasite (54, 55). Similar examples are found in both plant and wildlife disease systems (45). One major question has been whether the dilution effect operates for zoonotic diseases. An early metaanalysis suggested that it did not (56). However, a larger metaanalysis found that the dilution effect was as strong for zoonoses as for other types of diseases (9), a conclusion that was robust to criticisms from the authors of the earlier study (57, 58).

Despite abundant evidence for the dilution effect, the more general idea that biodiversity can reduce human disease risk has been controversial (47, 59), in large part because biodiversity was thought to be a source of new zoonotic pathogens via spillover (in the sense of Fig. 1*A*) (5, 8, 17, 47). The conflation of the effects of native biodiversity and the effects of the loss of biodiversity was also problematic, as described below. And much of the confusion arose because the process of zoonotic spillover was treated as distinct from the process of transmission once a zoonotic disease had already spilled over and become endemic.

**Fig. 4.**
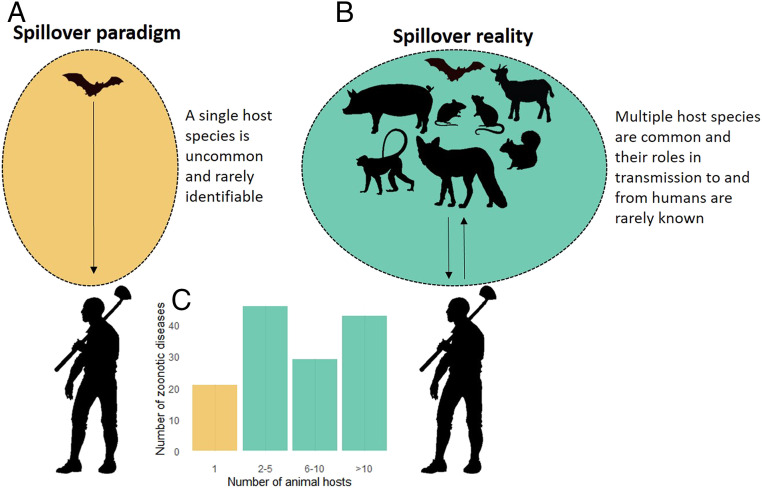
The paradigm and the reality for research on spillover of zoonotic pathogens into humans. (*A*) The paradigm emphasizes a single animal host species for a zoonotic pathogen and an original spillover event, though the event and the species are rarely identified. (*B*) In reality, most zoonotic pathogens have multiple host species whose specific roles in transmission to and from humans are rarely known. (*C*) The number of viral zoonotic diseases that have 1, 2 to 5, 6 to 10, or 11+ known animal host species other than humans. Plotted from data made available in supplementary materials from Johnson et al. (21); see caveats about these and similar data in *SI Appendix*.

Reconciling the effects of biodiversity on emergence and ongoing transmission requires acknowledging three critical points. First, most zoonotic pathogens are harbored by multiple host species (Fig. 4) that share the pathogen via cross-species transmission. Second, the emergence of a pathogen in a new host species, including humans, is just a special case of cross-species transmission. And finally, transmission from a current host to a potential new one, human or otherwise, is affected by the degree to which the current host actually transmits the pathogen (*SI Appendix*), which in turn is affected by the current host’s abundance, infectiousness, and infection prevalence (60). The majority of spillover studies have not included quantitative measures of transmission, relying instead on databases compiled from qualitative host–pathogen associations.

Plourde et al. (61) illustrate the potential of unifying spillover and transmission, and of relying on more quantitative metrics. They compiled a database of 330 zoonotic and nonzoonotic disease systems with pathogens that infect multiple host species and in which reservoir species (*SI Appendix*) are established or strongly implicated. Reservoir status is a more meaningful metric than the host–pathogen associations used in most spillover studies because it signifies transmission. Reservoirs for pathogens that cause diseases in humans were most commonly found in the Orders Rodentia (36%), Carnivora (25%), and Artiodactyla (21%) (61). Bats were reservoirs for only 8 (3%) of 261 disease systems, although 5 of these were high-priority zoonotic pathogens (based on an index of the number of publications about them). The most common reservoir hosts for zoonotic disease systems were commensal and domestic species, such as rats, dogs, cats, cattle, pigs, sheep, and goats.

Plourde et al. (61) found that reservoirs have significantly “faster” life histories—including shorter gestation periods, larger litters, lower neonate body mass, and younger age at sexual maturity—compared to nonreservoirs (62). Species with faster life histories have emerged as important from studies using other methods as well. Han et al. (22) found a similar pattern in rodents using host-pathogen associations for zoonotic diseases. Huang et al. (63) compared quantitative measures of transmission for three zoonotic diseases and found that hosts with the fastest life histories were more likely to transmit pathogens.

Why might life-history traits be related to the potential for a host to transmit a pathogen? A variety of studies suggest a tradeoff in investment in innate versus adaptive immunity, with shorter-lived species investing more in the former while longer-lived species invest more in the latter (64). Hosts that mount a weaker adaptive immune response (i.e., shorter-lived species) are thought to be more likely to maintain higher infectiousness, with an associated increase in transmission, as compared to hosts with stronger adaptive immunity. Previtali et al. (51) tested this idea by comparing immune responses among rodents that varied in life-history traits. They found that species with faster life histories mounted stronger innate immune responses, measured by bacterial killing capacity, compared to closely related species with slower life-history traits. These species also mounted weaker adaptive immune responses, measured by their antibody responses to a lipopolysaccharide challenge. Species with faster life histories were more likely to transmit *Borrelia burgdorferi*, the pathogen that causes Lyme disease in humans. Together, these results suggest a mechanism by which life-history strategies might be linked to the probability that a host species transmits a pathogen. Further evidence for a relationship between immune investment and host status is suggested by Gibb et al. (40), who found that mammal species that harbor a greater number of pathogen species are more abundant in human-impacted habitats. They conclude that there may be mammalian traits that impact both tolerance to human disturbance and tolerance to infection.

Quantifying differences between species in the ability to transmit zoonotic pathogens, and in the life-history and immunological traits associated with these abilities, facilitates the understanding of diversity–disease relationships. Because host species with fast life histories appear to be more likely to transmit pathogens, whether to species that are already hosts or to new hosts, including humans, zoonotic emergence, and transmission should be highest where hosts with fast life histories are abundant. Predicting the locations where these taxa thrive, and thus where transmission and emergence are likely, requires integrating what we know about biodiversity loss in natural ecosystems.

## Impacts of Biodiversity Loss on Zoonotic Diseases

When biodiversity is lost from ecological communities, the species most likely to disappear are large-bodied species with slower life histories (e.g., ref. 65), while smaller-bodied species with fast life histories tend to increase in abundance (e.g., ref. 66). Recent research shows that fast-lived species are more likely to transmit zoonotic pathogens (61). Together, these processes are likely to lead to increases in the abundance of zoonotic reservoirs when biodiversity is lost from ecological systems.

Supporting these predictions, Johnson et al. (21) found that mammalian hosts of zoonotic viruses are less likely to be of conservation concern (Fig. 3). For both mammals and birds, Gibb et al. (40) linked land-use changes caused by humans to increases in the abundance of zoonotic host species. They also report that declines in diversity of nonhosts are correlated with increases in the abundance and diversity of hosts, but they do not report whether there are net changes in overall biodiversity. A rich literature on infectious diseases of wildlife, livestock, and plants demonstrates increased pathogen transmission when biodiversity is lost from some ecological communities (9, 53), supporting the generality of this relationship across nonzoonotic disease systems as well.

## Concluding Remarks

Recent research has begun to reconcile the perceived conflict between the beneficial effects of maintaining natural biodiversity, through the dilution effect, with its purported costs as a source for new human pathogens. Cross-species transmission of pathogens to humans is a special case of an ongoing process that occasionally results in successful spillover into a new species, human or otherwise. Those pathogens that do spill over to infect humans—zoonotic pathogens—appear to be most likely to come from particular taxa, which often proliferate as a result of human impacts.

While the taxonomic group determined to be most responsible for zoonotic pathogens varies between studies, certain taxa—rodents, bats, primates, (cet)artiodactyls, and carnivores—consistently arise as the most important of the mammals. Given this knowledge, it is time to explore which metrics of host contributions are most useful for predicting and preventing spillover (*SI Appendix*) rather than continuing to debate the prime importance of one taxon or another. Because most pathogens that jump to humans have multiple nonhuman hosts (Fig. 4), it is time for the scientific community to at last put to rest the myth of there being “a reservoir” for most pathogens (67). Furthermore, domesticated and commensal species from across these taxonomic categories often serve as critical hosts, whether as the original source of a pathogen or as a secondary host with elevated contact with humans. It is time to focus on rigorous assessments of the relative contributions of changes in human behavior versus changes in ecological communities, and of their synergies.

Going forward, we need to acknowledge that the “total host diversity” model (Fig. 1*A*) is no longer adequate or appropriate given what we have learned over the past decade about the emergence and transmission of zoonotic pathogens. Instead, we need to focus on gathering and analyzing data that are relevant to transmission—data on the diversity, abundance, and capacity to transmit of the taxa that actually share zoonotic pathogens with us (Fig. 1*C*)—rather than continuing to succumb to the allure of readily available low-quality data and overly simple conceptual models. Certainly, we need more data on the effects of the abundance of hosts on zoonotic emergence, which will allow us to more confidently evaluate the “zoonotic host diversity” model versus the “zoonotic host diversity and abundance” model (Fig. 1). And we need to disentangle the effects of the innate characteristics of host species (such as their immune strategies, resilience to disturbance, and habitat preferences) from the effects of human behaviors (including management of domesticated species), which affect contact rates and other important factors in transmission.

Efforts to understand the role of biodiversity in zoonotic diseases should also clearly distinguish between the effects of natural levels of biodiversity and the effects of changes to this diversity, for example, through human impacts (53). Geographic comparisons through large-scale correlational studies (based on the “total host diversity” model in Fig. 1) have tended to report a weak but positive effect of mammal species richness on zoonotic diseases, but these studies show much stronger positive correlations with other factors, such as human population density (e.g., refs. 5 and 17). In contrast, biodiversity loss has been shown to often increase the risk of zoonotic diseases, for example, through the dilution effect (9). This distinction takes on particular importance in the context of policy and management because biodiversity loss can be addressed by human actions, however difficult this might be, while latitudinal gradients in diversity, for example, cannot be. Determining how different anthropogenic impacts (e.g., habitat conversion, climate change, overharvesting) affect zoonotic hosts is an important area of future research and has great promise, as recent research has demonstrated (21, 40).

Many other questions remain as well, including how best to gather data on the relative contributions of hosts for zoonotic pathogens and whether restoring biodiversity to areas degraded by human impacts reduces the abundance of zoonotic hosts. Understanding the factors that contribute to zoonotic disease emergence and transmission has never been more urgent, nor have the costs of failing to address them ever been more apparent.

## Supplementary Material

Supplementary File

## Data Availability

All study data available from the supplemental material in Johnson et al. (21).
