# Supplementary material for: Impacts of biodiversity and biodiversity loss on zoonotic diseases
Source: Proc Natl Acad Sci U S A. 2021 Apr 5;118(17):e2023540118. doi: 10.1073/pnas.2023540118 (PMC8092607; doi:10.1073/pnas.2023540118)
Supplement: Supplementary File [file pnas.2023540118.sapp.pdf]

PNAS
www.pnas.org

Supplementary Information for
Impacts of biodiversity and biodiversity loss on zoonotic diseases

Felicia Keesing and Richard S. Ostfeld

Felicia Keesing
Email: keesing@bard.edu

This PDF file includes:

Supplementary information text
Supplementary references

Supplementary Information Text

Hosts, reservoirs, and transmission. Databases of zoonotic hosts are compiled with evidence that a particular species, such as a rodent or a bat, is associated with a particular pathogen. These host-pathogen associations are often determined by evidence of seroconversion, indicating that a host has previously mounted an immune response after exposure to a pathogen. In some cases, genetic material from the pathogen has been isolated from the host species. That association might be recorded in a genomic database, or in the scientific literature. In some cases, there is evidence that a host species can shed the pathogen in bodily fluids or into the environment, or the host might be known to infect feeding vectors (such as mosquitoes, biting flies, or ticks). Any of these pieces of evidence would be sufficient to determine that an animal is a ***host*** of a particular pathogen, because the host can harbor the pathogen. Seroconversion is generally considered weak evidence of infection, because antibiodies can be produced without a lasting infection and can persist after any infection is cleared.

By definition, all zoonotic pathogens are hosted by at least two species, humans and at least one other vertebrate animal. Most zoonotic pathogens are hosted by more (Figure 4) (1–3). Typically, these hosts are not all equally capable of transmitting infection to known or to new hosts. Some hosts that are infected might not transmit the pathogen at all. A host species that transmits the pathogen to other hosts, and at rates sufficient to maintain its presence in a disease system, is often called a **reservoir**, though the precise definition of a reservoir varies (4–8).

Species that can transmit a pathogen often vary in the degree to which they do so, a capacity referred to as their **host competence** or **reservoir competence** (9–11), but this information is difficult to obtain (12) and is quantified for relatively few disease systems. In one well-studied ecosystem in upstate New York, USA, researchers have estimated the relative abilities of a number of host species to transmit three different zoonotic pathogens, each of which is carried by the same tick vector, the blacklegged tick (*Ixodes scapularis*) (13–18). For the most prevalent of these pathogens, *Borrelia burgdorferi*, which causes Lyme disease in humans, the **most**

competent host is the white-footed mouse, *Peromyscus leucopus*, with individual mice infecting ~90% of the uninfected tick vectors that feed on them. Many host species infect only a small percentage of feeding ticks, and are thus considered poor reservoirs.

Identifying zoonotic hosts. Many studies have reported the animal taxa likely to be sources of zoonotic pathogens. Differences in conclusions among these studies depend in part on the nature of their underlying datasets, including their timespan and whether they include all types of zoonotic pathogens, or just a subset such as viruses. The quality of the evidence used to determine whether a species is a host of a particular pathogen also varies among studies, as described above. Few require evidence of the ability of a host to actually transmit a pathogen (but see (8, 19)).

The use of serological and nucleic acid data in wild or domesticated animals is complicated by the fact that many zoonotic pathogens can also be transmitted from humans to other vertebrate animals. Databases of vertebrate species associated with a pathogen typically do not differentiate which species were sources of pathogen transmission to humans, and which were recipients of transmission from humans (19).

Differences in conclusions about zoonotic hosts also arise when researchers emphasize the overall effect of a taxon versus whether that effect differs from *a priori* expectations. For example, rodents are often identified as a major source of zoonotic spillover (20–23). Some studies minimize the significance of this result because rodents are the most diverse Order of mammals and thus would be expected to host more pathogens when considered as a group. Instead, these studies tend to emphasize taxa that are a *disproportionate* zoonotic source, but studies disagree on which taxa are disproportionately represented. For example, Luis et al. (23) identify bats as being disproportionately represented, while Mollentze & Streicker (19) argue that their representation is proportionate.

SI References

1. A. B. Pedersen, S. Altizer, M. Poss, A. A. Cunningham, C. L. Nunn, Patterns of host specificity and transmission among parasites of wild primates. *Int. J. Parasitol.* **35**, 647–57 (2005).
2. H. K. Alexander, T. Day, Risk factors for the evolutionary emergence of pathogens. *J. R. Soc. Interface* **7**, 1455–74 (2010).
3. S. Cleaveland, M. K. Laurenson, L. H. Taylor, Diseases of humans and their domestic mammals: pathogen characteristics, host range and the risk of emergence. *Philos. Trans. R. Soc. Lond. B. Biol. Sci.* **356**, 991–9 (2001).
4. M. Viana, *et al.*, Assembling evidence for identifying reservoirs of infection. *Trends Ecol. Evol.* **29**, 270–279 (2014).
5. D. T. Haydon, S. Cleaveland, L. H. Taylor, M. K. Laurenson, Identifying reservoirs of infection: A conceptual and practical challenge. *Emerg. Infect. Dis.* **8**, 1468–1473 (2002).
6. J. O. Lloyd-Smith, *et al.*, Epidemic dynamics at the human-animal interface. *Science* **326**, 1362–1367 (2009).
7. R. W. Ashford, When is a reservoir not a reservoir? *Emerg. Infect. Dis.* **9**, 1495–1496 (2003).
8. B. T. Plourde, *et al.*, Are disease reservoirs special? Taxonomic and life history characteristics. *PLoS One*, 1–23 (2017).
9. L. B. Martin, S. C. Burgan, J. S. Adelman, S. S. Gervasi, Host competence: An organismal trait to integrate immunology and epidemiology. *Integr. Comp. Biol.* **56**, 1225–1237 (2016).
10. S. S. Gervasi, D. J. Civitello, H. J. Kilvitis, L. B. Martin, The context of host competence: A role for plasticity in host-parasite dynamics. *Trends Parasitol.* **31**, 419–425 (2015).
11. T. E. Merrill, P. T. Johnson, Towards a mechanistic understanding of competence: a missing link in diversity–disease research. *Parasitology*, 1–12 (2020).
12. D. J. Becker, S. Seifert, C. Carlson, Beyond infection: integrating competence into reservoir host prediction. *Trends Ecol. Evol.* (in press).
13. F. Keesing, *et al.*, Reservoir competence of vertebrate hosts for *Anaplasma phagocytophilum*. *Emerg. Infect. Dis.* **18**, 2013–2016 (2012).
14. M. H. Hersh, M. Tibbetts, M. Strauss, R. S. Ostfeld, F. Keesing, Reservoir competence of wildlife host species for *Babesia microti*. *Emerg. Infect. Dis.* **18**, 1951–1957 (2012).
15. F. Keesing, *et al.*, Prevalence of human-active and variant 1 strains of the tick-borne pathogen *Anaplasma phagocytophilum* in hosts and forests of eastern North America. *Am. J. Trop. Med. Hyg.* **91**, 302–309 (2014).
16. J. L. Brunner, K. LoGiudice, R. S. Ostfeld, Estimating reservoir competence of *Borrelia burgdorferi* hosts: prevalence and infectivity, sensitivity, and specificity. *J. Med. Entomol.* **45**, 139–47 (2008).
17. R. Ostfeld, *Lyme disease: the ecology of a complex system* (Oxford University Press, 2010) (November 5, 2013).
18. F. Keesing, *et al.*, Hosts as ecological traps for the vector of Lyme disease. *Proc. Biol. Sci.* **276**, 3911–9 (2009).
19. N. Mollentze, D. G. Streicker, Viral zoonotic risk is homogenous among taxonomic orders of mammalian and avian reservoir hosts. *Proc. Natl. Acad. Sci.* **117**, 9423–9430 (2020).
20. K. J. Olival, *et al.*, Host and viral traits predict zoonotic spillover from mammals. *Nature* **546**, 646–650 (2017).
21. B. A. Han, A. M. Kramer, J. M. Drake, Global patterns of zoonotic disease in mammals. *Trends Parasitol.* **32**, 565–577 (2016).
22. C. K. Johnson, *et al.*, Global shifts in mammalian population trends reveal key predictors of virus spillover risk. *Proc. R. Soc. B* **287**, 20192736 (2020).
23. A. D. Luis, *et al.*, A comparison of bats and rodents as reservoirs of zoonotic viruses: are bats special? *Proc. Biol. Sci.* **280**, 20122753 (2013).
